# CX3CL1 Overexpression Prevents the Formation of Lung Metastases in Trastuzumab-Treated MDA-MB-453-Based Humanized Tumor Mice (HTM)

**DOI:** 10.3390/cancers13102459

**Published:** 2021-05-18

**Authors:** Anja Kathrin Wege, Tobias F. Dreyer, Attila Teoman, Olaf Ortmann, Gero Brockhoff, Holger Bronger

**Affiliations:** 1 Department of Gynecology and Obstetrics, University Cancer Center Regensburg, 93053 Regensburg, Germany; ateoman@caritasstjosef.de (A.T.); olaf.ortmann@ukr.de (O.O.); gero.brockhoff@ukr.de (G.B.); 2 Department of Gynecology and Obstetrics, Technical University of Munich, 81675 Munich, Germany; tobias.dreyer@tum.de (T.F.D.); holger.bronger@tum.de (H.B.)

**Keywords:** CX3CL1, humanized tumor mice (HTM), trastuzumab, HER2, breast cancer, ADAM10, ADAM17

## Abstract

**Simple Summary:**

In about 15–18% of breast cancers the HER2 gene is amplified, which allows an anti-HER2 treatment. However, about 50% of HER2-positive patients experience de novo or acquired resistance to the antibody-based therapy with trastuzumab. Therefore, the identification of predictive markers for therapy success and novel combination strategies is needed. Here we explored the impact of CX3CL1 on trastuzumab treatment efficiency and immunological mechanism involved in a humanized tumor mouse model. Trastuzumab treatment showed pronounced efficiency in CX3CL1 overexpressing cancer cells compared to low expressing cells preventing lung metastasis, while the administration of CX3CL1 shedding inhibition did not cause an enhanced treatment effect. Moreover, the application of shedding inhibitors to CX3CL1 overexpression tumors resulted in a slightly enhanced tumor growth. Therefore, the presence of CX3CL1 might predict a pronounced response to trastuzumab therapy in patients and should be investigated in a large cohort of HER2^+^ patients.

**Abstract:**

CX3CL1 is a multifunctional chemokine that is involved in numerous biological processes, such as immune cell attraction and enhanced tumor immune cell interaction, but also in enhancing tumor cell proliferation and metastasis. The multifarious activity is partially determined by two CX3CL1 isoforms, a membrane-bound and a soluble version generated by proteolytic cleavage through proteases. Here, we investigated the impact of CX3CL1 overexpression in MDA-MB-453 and SK-BR-3 breast cancer cells. Moreover, we evaluated the therapeutic capacity of Matrix-Metalloproteinases-inhibitors TMI-1 and GI254023X in combination with the anti-HER2 antibody trastuzumab in vitro and in vivo. TMI-1 and GI254023X caused a reduced shedding of CX3CL1 and of HER2 in vitro but without effects on tumor cell proliferation or viability. In addition, trastuzumab treatment did not retard MDA-MB-453 cell expansion in vitro unless CX3CL1 was overexpressed upon transfection (MDA-MB-453^CX3CL1^). In humanized tumor mice, which show a coexistence of human tumor and human immune system, CX3CL1 overexpression resulted in a slightly enhanced tumor growth. However, trastuzumab treatment attenuated tumor growth of both MDA-MB-453^CX3CL1^ and empty vector transfected MDA-MB-453 transplanted mice but showed enhanced efficiency especially in preventing lung metastases in CX3CL1 overexpressing cancer cells. However, TMI-1 did not further enhance the trastuzumab treatment efficacy.

## 1. Introduction

Breast cancer (BC) is the most frequent malignant disease in women in the Western world [1]. About 15–18% of the breast cancers belong to the HER2-positive (HER2^+^) subtype that intrinsically exhibits a poor prognosis. However, therapy with the monoclonal, humanized anti-HER2 antibody trastuzumab and other anti-HER2 therapeutic agents considerably improved outcome of HER2^+^ BC in the past two decades, although about 50% of patients still experience de novo or acquired resistance [2]. Thus, refining and enhancing the anti-HER2 targeting, e.g., by a combined target-specific approach, is expected to improve the clinical outcome in trastuzumab-treated patients. 

Regardless of an anti-HER2 treatment, tumor immune cell infiltration (TIL) is associated with improved outcome of the patients in particular in HER2^+^, but also in triple-negative BC [3,4,5]. An antibody therapy such as trastuzumab impairs tumor cell growth by direct cellular mechanisms, but also triggers NK cells by a mechanism known as antibody-dependent cellular cytotoxicity (ADCC [6,7]). Therefore, the attraction of human immune cells into the tumor microenvironment seems to be essential for a pronounced therapeutic success.

CX3CL1 is a multifunctional chemokine, which is involved in a variety of tumor-relevant processes, such as immune cell attraction and enhanced tumor–immune cell interaction on the one hand, and in enhancing tumor cell proliferation, invasion, angiogenesis and increased metastasis on the other hand [8]. CX3CL1 exists in two different variants, the membrane-located form and the proteolytically cleaved and soluble domain. CX3CL1 exhibits multiple cleavage sites leading to coexisting, differentially shed products [9]. The soluble CX3CL1, also called Fractalkine, has the capacity to attract different immune effector cells (e.g., T and NK cells) into the tumor environment [10,11,12]. However, it might also guide the CX3CR1-positive tumor cells out of the primary tumor towards pre-metastatic niches, for instance towards the bone marrow (bm) [13]. The overexpression of the corresponding receptor CX3CR1 has been associated with a higher tendency of BC cells to spread to the skeleton [14] or to the brain [15]. Soluble CX3CL1 might also bind to CX3CR1 expressing tumor cells and trigger tumor cell proliferation. This has been first described in human smooth muscle cells mediated via the transactivation of the epidermal growth factor receptor (EGFR; HER1)-dependent signaling by CX3CL1 [16]. Later on, Tardàguila and colleagues confirmed this CX3CL1 transactivation of the EGFR pathway for BC using a HER2/neu transgenic mouse model [17]. In contrast to the soluble variant, the membrane located CX3CL1 with its physiological function as adhesion molecule [18] promotes stronger adhesion of leucocytes to CX3CR1-positive cells, and therefore, might enhance T cell killing or NK cell mediated ADCC on trastuzumab coated BC tumor cells [19].

The proteolytic cleavage (i.e., the extracellular shedding) of the membrane-bound CX3CL1 to the soluble form is mainly mediated by metalloproteases ADAM10 (A Disintegrin and metalloproteinase domain-containing protein) [20,21] and ADAM17, also known as tumor necrosis factor-α (TNFα)-converting enzyme (TACE) [19,21]. Different inhibitors for ADAMs are available and a number of them are being currently subjected to clinical trials. The inhibitor TMI-1 blocks ADAM17, ADAM10 and other MMPs and has been shown to be effective on triple negative and HER2-overexpressing breast cancer cells. GI254023X preferentially impairs ADAM10 activity [21], but to a lesser extend also inhibits other MMPs as well as ADAM17.

The numerous activities of soluble and membrane-bound CX3CL1 might explain ostensibly inconsistent reports on the impact of this chemokine on the course and outcome of BC disease. For example, while Park et al. found a correlation between high CX3CL1 expression with a good prognosis and increased immune cell infiltration [22], others reported a poor outcome of BC patients that was associated with CX3CL1-positive tumor cells [23].

Here, we evaluated the anti-tumor activity of a combined trastuzumab and TMI-1/GI254023X treatment in the context of CX3CL1 expression. We tested the effects of both ADAM inhibitors in vitro (MDA-MB-453 and SK-BR-3) and further applied TMI-1 in combination with trastuzumab in CX3CL1-expressing (MDA-MB-453^CX3CL1^) and control (MDA-MB-453^empty^)-based HTM. HTM enable the assessment of treatment strategies under human-like conditions, since they come with a coexisting human immune system and human tumor growth as a result of the transplantation of human hematopoietic stems cells and human BC cells into immunodeficient NSG mice [24,25,26,27].

## 2. Materials and Methods

### 2.1. BC Cell Lines and Treatments

MDA-MB-453 and SK-BR-3 used in this study were obtained from the American Type Culture Collection (ATCC, LGC Standards, Wesel, Germany) and authentication was confirmed in November 2017 and June 2018 from the German DSMZ Leibniz Institute by a nanoplex PCR-based STR profiling. Cells were transfected as previously described [28]. Briefly, stable transfection was performed by using three µg of vector DNA (pCMV 6 mCX3CL1, pCMV 6 hCX3CL1, pCMV-6-Entry, Origene) and 6 µL of Lipofectin (Thermo Fisher Scientific, Darmstadt, Germany). Transfected cells were selected with Neomycin (Thermo Fisher Scientific, Darmstadt, Germany) for 14 days and positive clones used for in vitro and in vivo testing after CX3CL1 measurement in an ELISA system (Human CX3CL1/Fractalkine DuoSet ELISA, R&D Systems, Wiesbaden, Germany) according to the manufacturer’s protocol. Cells were incubated and expanded in Dulbecco’s modified Eagle’s medium (DMEM), supplemented with 10% fetal calf serum (FCS) (both PAA Laboratories, Pasching, Austria) and 0.5 mg/mL G418 (Geneticin Sulphate, Thermo Fisher Scientific) under standard cell culture conditions. Cells were treated in vitro with trastuzumab (5 µg/mL), ADAM Inhibitor TMI-1 (10 µM; PZ0336, Sigma-Aldrich, Darmstadt, Germany), and GI254023X (5 µM; SML0789; Sigma-Aldrich, Germany) for 48 h. Untreated cells served as control samples. Supernatant from cell culture and serum from MDA-MB-453 transplanted Humanized Tumor Mice (HTM) were tested for CX3CL1 (Human CX3CL1/Fractalkine DuoSet ELISA, R&D Systems, Germany) and soluble HER2 (Human ErbB2/Her2 ELISA, R&D Systems, Germany) according to the manufacturer’s protocol. For the anti-HER2–trastuzumab competition assay, cells were stained for 60 min on ice with increasing trastuzumab concentrations (0–10 µg/mL), washed and afterward incubated for 20 min with the anti-HER2 FITC antibody (clone 24D2, BioLegend, San Diego, CA, USA).

### 2.2. Western Blotting 

Tumor cells were directly lysed in the cell culture flasks with cell-lysis buffer (Cell Signaling, Danvers, MA, USA). The concentration of total protein was determined using the BCA Protein Assay Kit (Pierce, Thermo Fisher Scientific, Rockord, IL, USA). Twenty µg of protein samples were separated by a 10% SDS-PAGE and blotted onto a 0.2 µm PVDF membrane by semi-dry wet blotting. Afterwards, the membrane was blocked in 5% BSA powder in TBS-T for one hour at room temperature. Incubation with the primary antibody anti-ADAM 10 (1:1000; Cell Signaling, Danvers, MA, USA) and anti-β-actin (1:5000; Sigma-Aldrich, Taufkirchen, Germany) was performed in TBS-T (supplemented with in 2.5% BSA) overnight (4 °C). Secondary horseradish peroxidase (HRP)-conjugated goat-anti rabbit IgG antibody (1:2000; Cell Signaling, Danvers, MA, USA) was incubated for one hour at room temperature. Detection was performed using SuperSignal™ West Pico PLUS Chemiluminescent Substrate (Thermo Fisher Scientific, Darmstadt, Germany) and analyzed with the ImageQuant LAS 4000 mini (GE Healthcare, Buckinghamshire, UK).

### 2.3. Mice

NOD.Cg-Prkdc*^scid^* Il2rγ^tm1Wjl/^SzJ (NSG) mice were obtained from Jackson Laboratories and bred and kept in a specialized pathogen-free facility at the University of Regensburg. Humanized tumor mice were generated as previously described [24,25,26]. Briefly, newborn mice were irradiated (1 Gy) and, 3 h later, transplanted with ~1–2 × 10^5^ human CD34^+^ cells isolated from umbilical cord blood (CB) using immunomagnetic beads (Miltenyi Biotech, Bergisch Gladbach, Germany). At the age of 8 weeks, humanized mice were bled and the human reconstitution status (% CD45, CD3, CD19, CD33) were analyzed. Humanized mice with >15% CD45/live cells were transplanted orthotopically with 50,000 (pilot study) and 1 × 10^6^ MDA-MB-453^CX3CL1^ or MDA-MB-453^empty^ in medium without substitutions. Importantly, mice transplanted with the same CB sample were split into different treatment and control groups. Treatment started when the tumor measured 5 mm in diameter with trastuzumab (5 mg/kg/week i.p.) or ADAM Inhibitor TMI-1 (100 mg/kg twice a week i.p.) for three weeks and analyzed 7 days after the last treatment (Supplementary Figure S1). 

### 2.4. Ethic Statements

The local veterinary authorities of the district government of Lower Franconia and Upper Palatinate (Bavarian region) approved all animal work (permission no. 55-2-2532-2-381). Cord blood samples were taken based on the approval given by the Ethics Committee of the University of Regensburg (permission no. 16-101-0179). All patients included in the study provided written informed consent.

### 2.5. Immunohistochemistry 

Tissue specimens (tumor, spleen, liver, brain and lung) were prepared as previously described [24,25,26]. Briefly, samples were fixed with 4% formalin and embedded in paraffin. Four µm slides were prepared, deparaffinized and stained with anti-HER2 rabbit polyclonal A0485, anti-CK18 (clone DC 10), anti-CD20 (Clone L26) and anti-CD68 (Clone KP1) from Dako GmbH (Jena, Germany) and anti-CD4 (clone SP35) and anti-CD8 (clone SP57) from Ventana (Tucson, AZ, USA), Staining was performed automatically on a Ventana Nexes autostainer (Ventana, Tucson, AZ, USA) by using the streptavidin-biotin-peroxidase complex method and 3,3′-diaminobenzidine. Histological specimens were imaged with an AxioImager Z1 microscope (Zeiss, Oberkochen, Germany).

### 2.6. Flow Cytometry Analysis

Flow cytometry was performed on a FACSCanto II flow cytometer (BD Biosciences, San Jose, CA, USA) equipped with a blue (488 nm), violet (405 nm) and red (633 nm) laser and the data were analyzed with FACSDiva Software v7.0 (BD Biosciences,San Jose, CA, USA ). Unspecific binding was blocked by incubating the cells in 1% mouse serum for 10 min and appropriate mouse immunoglobulin antibodies were used as isotype controls for all staining. Organs (spleen, tumor and lung) were dissociated by passing the cells through 40 μm cell strainer (BD Bioscience). Bone marrow (BM) cells were collected from the femur by clipping the ends and flushing the bone cavity with 10 mL PBS using a syringe with a 27 G needle (BD Bioscience).

(a) Tumor cell phenotyping: Samples were stained using the following antibodies: anti-HER2-FITC (clone 24D2, BioLegend), anti-CD47-PE (clone B6H12, BD Biosciences), anti-CD44 Pe-Cy7 (clone G44-26, BD Biosciences), anti-c-MET APC (clone # 95106, R&D Systems), anti-CD24 APC Vio770 (clone 32D12, Miltenyi Biotec, Bergisch Gladbach, Germany) anti-CD45 BV510 (clone H130, BioLegend, San Diego, CA, USA) and anti-EPCAM BV421 (clone 9C4, BioLegend).
(b) Human immune cell reconstitution in spleen and tumor were analyzed with anti-CD3-FITC (clone UCHT1), anti-CD19-PE (clone HIB19) and anti-CD45-APC (clone HI30) from BD Bioscience and anti-CD33-PerCPCy5.5 (clone WM53) from BioLegend.
(c) T cell phenotyping was analyzed by anti-CD3-FITC (clone UCHT1, BD Bioscience), anti-CD127-PE (clone A019D5, BioLegend), anti-CD27-PeCy7 (clone O323, eBioscience (Thermo Fisher Scientific), Darmstadt, Germany), anti-PD-1-AF647 (clone EH12.2H7, BioLegend), anti-CD4-APC-H7 (clone SK3, BD Bioscience), anti-CD8a-BV510 (clone RPA-T8, BioLegend) and anti-CD45RA-BV421 (clone HI100, BioLegend)
(d) NK cells were characterized by anti-NKp46-APC (clone 9-E2, BD Bioscience), anti-CD16-PE (clone 3G8, BioLegend) and anti-CD56-Horizon™V450 (clone B159, BD Bioscience).
(e) CX3CR3 expression on immune cells and tumor cells were analyzed by anti-HER2-FITC (clone 24D2, BioLegend), anti-CD8-PE (clone HIT8a, BD Bioscience), anti-CD45-APC (clone HI30, BD Bioscience), anti-CXCR3-BV421 (clone 2A9-1, BioLegend), anti-CD4-APC-H7 (clone SK3, BD Bioscience), anti-CD19-BV510 (clone HIB19, BioLegend) and anti-NKp46-APC (clone 9-E2, BD Bioscience). CX3CR1 expression on tumor cells were performed by anti-CXCR3-BV421 (clone 2A9-1, BioLegend).
(f) Proliferation assessment: Cells were harvested by trypsinization and washed twice with PBS, followed by fixation and permeabilization with cooled MeOH (70%) overnight. Afterward the cells were washed and incubated for 20 min in the presence of RNase (Roche Molecular Systems) at 37 °C and finally stained with 1 μg/mL DAPI prior to analysis. Cell doublets, aggregates and ¬debris were excluded by pulse processing and DNA histograms of the gated population were plotted on a linear scale. Cell cycle fractions (% of cells in G0/G1-, S- and G2/M-phase) were analyzed using the ModFit LT 3.2 software (Verity Software House, Topsham, ME, USA).
(g) Annexin V-FITC/DAPI Assay: Cells were cultured for 72 h in 10% FCS/DMEM and treated for 48 h with trastuzumab, ADAM Inhibitor TMI-1 and GI254023X. Untreated cells served as negative control. Cells were harvested by trypsinization without discarding the supernatant and stained using the TACS Annexin-V-FITC Apoptosis Detection Kit (ImmunoTools, Friesoythe, Germany) according to the manufacturer’s instructions.

### 2.7. Statistical Analyses

All results are shown as mean ± SEM. All reported *p*-values were two-sided. *p*-values less than 0.05 were considered significant. For groupwise comparison, a one-way or two-way analysis of variance (ANOVA) with Dunnett’s post-hoc test or Tukey’s multiple comparisons were applied and the tests are indicated in each figure and table legend. All statistical analyses were performed using GraphPad Prism (Ver. 6, GraphPad Software, La Jolla, CA, USA).

### 2.8. List of Abbreviations

BC: breast cancer, BM: bone marrow, CB: cord blood, CTL: cytotoxic T lymphocytes, HER2: human epidermal growth factor receptor 2, HSC: hematopoietic stem cells, HTM: humanized tumor mice, mAbs: monoclonal antibodies, NK: natural killer.

## 3. Results

### 3.1. TMI-1 and GI254023X Inhibit CX3CL1 and HER2 Shedding In vitro without Affecting Tumor Cell Proliferation

MDA-MB-453 belong to the HER2^+^ subentity with a moderate HER2 gene amplification (HER2/CEN17 ratio of 2.15) and HER2 overexpression [29]. These cells only express very low levels of CX3CL1, which cannot be further elevated by inflammatory cytokines such as TNF-α or IFN-γ [28]. For this reasons, MDA-MB-453 was chosen for the transfection experiments to identify the impact of CX3CL1 expression on tumor growth, metastases formation and treatment efficiency of trastuzumab and ADAM inhibitors. Transfection efficiency was determined by ELISA directly upon transfection and before each in vitro and in vivo experiment (MDA-MB-453^CX3CL1^: 97 ng/mL ± 20.95 SEM, *n* = 6; MDA-MB-453^empty^: 0.58 ng/mL ± 0.11 SEM, *n* = 6; *p* = 0.0010). 

**Figure 1:**
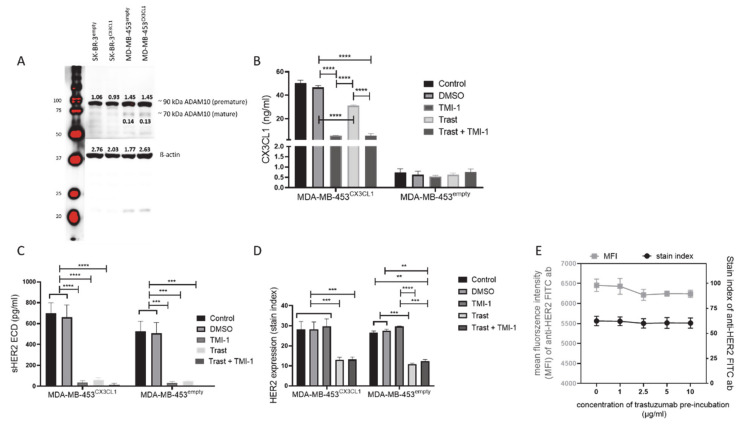
CX3CL1 and HER2 shedding and expression in trastuzumab and TMI-1 treated MDA-MB-453 tumor cells. MDA-MB-453 and SK-BR-3 cells were stably transfected with human Cx3CL1 (Cx3CL1^+^) or vector control (empty). (**A**) SK-BR-3/MDA-MB-453^CX3CL1^ and SK-BR-3/MDA-MB-453^empty^ were stained for ADAM 10 expression using western blot technology. MDA-MB-453^CX3CL1^ and MDA-MB-453^empty^ were cultured with a total concentration of 5 µg/mL trastuzumab (Trast) and 10 µM TMI-1 for 48 h and supernatants were analyzed for CX3CL1 (**B**) or HER2 extracellular domain (HER2 ECD) (**C**) in comparison to DMSO treated control cells by ELISA. (**D**) HER2 expression intensity of the treated and untreated MDA-MB-453 cells was analyzed by flow cytometry and the stain index (MFI = mean fluorescence intensity; stain index = (MFI_Her2_ − MFI_Isotype_)/(2 (×) SD_Isotype_)) was calculated. (**E**) MFI and the stain index of the anti-HER2 staining after 60 min of trastuzumab pre-incubation (with increasing concentration: 0–10 µg/mL) on ice are displayed. All experiments were performed in triplicate. Data are shown as mean ± SEM and Tukey’s multiple comparisons test was applied and significances are indicated (**p < 0.01; *** *p* < 0.001; **** *p* < 0.0001).

Proteolytic shedding of the membrane bound CX3CL1 is mainly mediated by metalloproteases ADAM10, which is expressed in SK-BR-3 and MDA-MB-453 (Figure 1A), showing the premature ADAM10 with 90 kDa and to a lower extend the mature form of ADAM10 with 60–70 kDa [30]. TMI-1 treatment resulted in significantly reduced CX3CL1 shedding from CX3CL1 transfected MDA-MB-453, whereas no reduction (and nearly no CX3CL1 concentration) could be observed in MDA-MB-453^empty^ cells upon TMI-1 treatment (Figure 1B). However, both TMI-1 as well as trastuzumab treatment impaired HER2 shedding in MDA-MB-453^empty^ and in MDA-MB-453^CX3CL1^ cells (Figure 1C). Similar results were obtained in SK-BR-3, but due to the overall lower CX3CL1 expression upon transfection (SK-BR-3^CX3CL1^: 14.18 ng/mL ± 7.3 SEM, *n* = 3; SK-BR-3^empty^: 1.89 ng/mL ± 0.79 SEM, *n* = 3; *p* = 0.0446), there was no significant difference in CX3CL1 in the supernatant upon TMI-1 treatment (Supplementary Figure S2A), but a similar tendency as shown for MDA-MB-453 cells. 

Interestingly, TMI-1 treatment did significantly reduce HER2 shedding of MDA-MB-453 (HER2 ECD detection in ELISA; Figure 1C) and SK-BR-3 (Supplementary Figure S2B), but the total HER2 expression on the cell surface using flow cytometry in both cell lines was not affected (Figure 1D and S2C). By contrast, the application of trastuzumab attenuated the HER2 shedding but also significantly reduced the cell surface HER2 expression (Figure 1D and S2C). Surprisingly, SK-BR-3^empty^ cells showed a significant higher concentration of soluble HER2 ECD (Supplementary Figure S2B) and increased HER2 expression (Supplementary Figure S2C) compared to SK-BR-3^CX3CL1^. Titration of the anti-HER2 FACS antibody on ice using MDA-MB-453 tumor cells against increasing trastuzumab concentration (0–10 µg/mL) did not impede HER2 staining (Figure 1E), suggesting that there was no steric hindrance of trastuzumab and the anti-HER2 detection antibody but possibly enhanced internalization of HER2 by trastuzumab [31].

Analyses of cell viability revealed no apoptotic effect in any SK-BR-3 or MDA-MB-453 (Supplementary Figure S3A,B) tumor cells caused by TMI-1 or trastuzumab treatment. In addition, there were no significant changes in cell cycle progression of MDA-MB-453 cells (Supplementary Figure S3C,D), except for a significant reduction of S-phase in trastuzumab-treated MDA-MB-453^CX3CL1^ cells, which indicates a higher sensitivity of these cells to the antibody treatment (Supplementary Figure S3C). As expected, in SK-BR-3^CX3CL1^ (Supplementary Figure S2D) and SK-BR-3^empty^ (Supplementary Figure S2E), trastuzumab significantly reduced S-phase proportion, and thereby, increased G1-phase fraction without an (additional) effect by TMI-1 treatment.

Similar results were obtained by using the inhibitor GI254023X, which is supposed to mainly block ADAM10: a significant reduction of CX3CL1 (Supplementary Figure S4A, data shown for MDA-MB-453^CX3CL1^) and HER2 (Supplementary Figure S4B) shedding was revealed. The exposition to GI254023X also had no effect on cell number, cellular apoptosis (Supplementary Figure S4C,D) or cell cycle progression (Supplementary Figure S4E,F) of MDA-MB-453^empty^ and in MDA-MB-453^CX3CL1^ cells, respectively.

### 3.2. Trastuzumab Efficiently Blocked Tumor Growth with Enhanced Efficiency in CX3CL1 Overexpressing MDA-MB-453-Based HTM

Next, we investigated the effect of CX3CL1 expression and the impact of ADAM inhibitors using the humanized tumor mouse model (HTM), which allowed the investigation of the impact of CX3CL1 on tumor growth, metastatic spread and human immune cell modulation. The in vitro analysis revealed a rather low CX3CL1 expression in SK-BR-3 cells upon CX3CL1 transfection. Therefore, MDA-MB-453 cells were used to generate HTM for the in vivo study.

**Figure 2:**
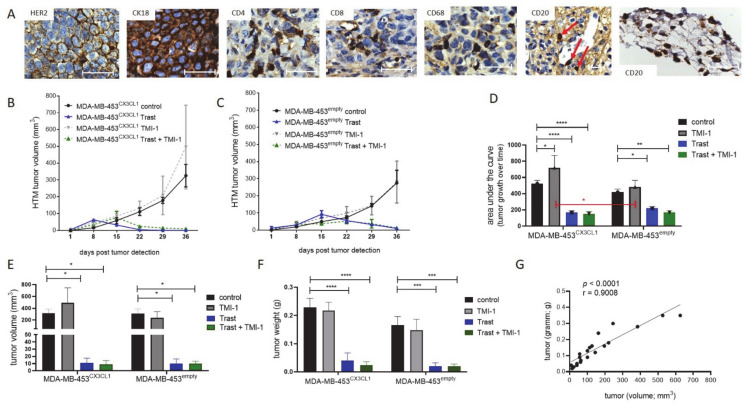
Treatment efficiency of trastuzumab and TMI-1 in MDA-MB-453-transplanted HTM. MDA-MB-453^CX3CL1^ and MDA-MB-453^empty^ were transplanted orthotopically in humanized NSG mice. Animals were randomized to receive trastuzumab (Trast), TMI-1, Trast+TMI-1 or DMSO control. (**A**) Immunhistological staining of tumor tissue (exemplarily shown for a one-time trastuzumab-treated HTM) for human HER2, cytokeratin 18, T helper (CD4), cytotoxic (CD8) T cells, macrophages (CD68) and B cells (CD20 in the periphery of the tumor and in the connective tissue). Red arrows indicate CD20^+^-stained cells. Tumor growth over time in MDA-MB-453^CX3CL1^. Scale bar represents 50 μm. (**B**) and MDA-MB-453^empty^ (**C**) in the different treatment groups is shown and summarized in (**D**). Tumor volume (**E**) and tumor weight (**F**) of the treated HTM and the correlation (**G**) between both parameters were analyzed (Pearson’s correlation coefficient *r* = 0.9008). (**D**–**F**) Data are shown for HTM, which achieved the end of the experiments (control *n* = 6; Trast *n* = 6, TMI-1 *n* = 5; TMI-1 + Trast *n* = 5) as mean ± SEM. Dunnett’s multiple comparison test (**D**–**F**; * *p* < 0.05; ** *p* < 0.01; *** *p* < 0.001, **** *p* < 0.0001) and Sidak’s multiple comparisons test (**D**, red bars; * *p* = 0.037) were applied and significances are indicated.

Histological staining of the primary tumors in HTM revealed variable HER2 expression levels in different tumor areas, but continuously high CK18 levels (Figure 2A), which proves human tumor origin (i.e., MDA-MB-453 cells). Immune cell infiltration was also detected in specific areas of the tumor tissue, showing infiltration of CD4^+^ T cells and CD68^+^ cells but rarely CD8^+^ T or B cells. B cells (CD20) were mostly found in the surrounding stroma (Figure 2A).

Nearly three weeks post tumor cell transplantation, tumors were detectable (mean days post transplant: 19.2 days ±1.8 SEM; *n* = 49 tumor) and monitoring of tumor growth was started. To ensure high CX3CL1 expression in vivo until the end of the experiments, CX3CL1 concentration in the tumors was analyzed in a pilot study. Constantly increased levels of CX3CL1 were detected after an average of 90 days upon transplantation in MDA-MB-453^CX3CL1^ (737.2 pg/µg ± 500 SEM; *n* = 5) versus MDA-MB-453^empty^ vector transfection (4.7 pg/µg CX3CL1 ± 1.4 SEM; *n* = 2) in HTM. Therefore, during the ~7-week treatment study, a high concentration of CX3CL1 in the tumor of MDA-MB-453^CX3CL1^ HTM was warranted.

Overall, there was an inhibition of tumor growth by trastuzumab treatment alone or in combination with TMI-1 in MDA-MB-453^CX3CL1^ and MDA-MB-453^empty^ HTM (Figure 2B,C). The analyses of tumor development over time (Figure 2D) revealed a significantly reduced tumor growth in trastuzumab-treated MDA-MB-453^empty^ (* *p* = 0.0201; Trast + TMI-1: ** *p* = 0.0082) and an even more pronounced difference upon trastuzumab treatment in MDA-MB-453^CX3CL1^ (**** *p* < 0.0001). Surprisingly, in MDA-MB-453^CX3CL1^, HTM TMI-1 showed a slightly enhanced tumor growth over time in comparison to control (Figure 2D; * *p* = 0.0475). There were no differences between MDA-MB-453^CX3CL1^ and MDA-MB-453^empty^ transplanted HTM detectable besides between TMI-1 treated mice (Figure 2D, red bar; *p* = 0.037). The treatment efficiency of trastuzumab (but not of TMI-1) is also reflected by the final tumor volume (Figure 2E) and the tumor weight (Figure 2F). Detailed information of each HTM is listed in the Supplementary Materials (Supplementary Table S1). As expected, there is a significant correlation between tumor weight and volume (Figure 2G; Pearson’s *r* = 0.9008).

### 3.3. Increased CD44 Expression In vivo on MDA-MB-453^CX3CL1^ and MDA-MB-453^empty^ Primary Tumor Cells

**Figure 3:**
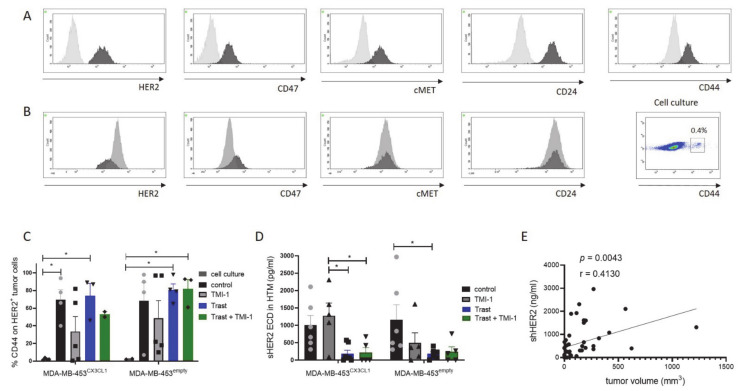
Tumor cell phenotyping and HER2 shedding detected in the serum of MDA-MB-453 HTM. Humanized NSG mice were transplanted with 1 × 10^6^ MDA-MB-453^CX3CL1^ and MDA-MB-453^empty^ and tumor cells were analyzed by flow cytometry. (**A**,**B**) Histogram of tumor cells stained with HER2, CD47, cMET, CD24 and CD44 are displayed (**A**: isotype control = grey; tumor isolated from HTM = black; **B**: cell culture = grey; tumor isolated from HTM = black). Dot plot shows the low percentage of CD44^+^ MDA-MB-453 tumor cells from the cell culture. (**C**) Percentage of CD44 expression on HER2+ tumor cells from cell culture compared with tumor cells isolated from the primary tumor of HTM are summarized. (**D**) Shed serum HER2 extracellular domain (sHER2 ECD) was detectable in the serum of HTM. Data are shown as mean ± SEM and significances were calculated using Tukey’s multiple comparisons test (*p < 0.05). (**E**) Graph outlines the correlation between sHER2 ECD and tumor volume (Pearson’s correlation coefficient *r* = 0.4130).

The characterization of tumor cell marker expression (exemplarily shown in Figure 3A) on EPCAM^+^ MDA-MB-453 cells isolated from the solid tumors of HTM revealed HER2, CD47, cMET, CD24 and CD44 expression. Tumor cells isolated from HTM (black histogram, Figure 3B) showed similar expression profiles of cMET and CD24 and slightly different HER2 and CD47 patterns (Figure 3B) compared to cell culture cells (grey histogram; Figure 3B). There were no differences between MDA-MB-453^CX3CL1^ and MDA-MB-453^empty^ cells or between treatment groups detectable when these markers were analyzed. However, CD44 expression on tumor cells in vivo (primary tumor) was significantly increased compared to cell culture cells (Figure 3C). More specifically, in vitro cells only exhibited a small subfraction of CD44^+^ tumor cells (Figure 3B, dot plot). CD44 expression also showed a variation between the treatment groups with the weakest expression in TMI-1-treated MDA-MB-453^CX3CL1^ and MDA-MB-453^empty^ HTM (Figure 3C, not significant). 

Shed HER2 extracellular domain (sHER2 ECD) could also be detected in the serum of HTM and revealed a significant reduction of sHER2 ECD in trastuzumab-treated (alone or in combination with TMI-1) HTM (Figure 3D). The amount of sHER2 ECD showed a moderate correlation to the corresponding tumor volume (Figure 3E; Pearson’s *r* = 0.4130; *p* = 0.0043).

### 3.4. Trastuzumab Treatment Significantly Reduced Lung Metastases in MDA-MB-453^CX3CL1^- but Not in MDA-MB-453^empty^-Based HTM

**Figure 4:**
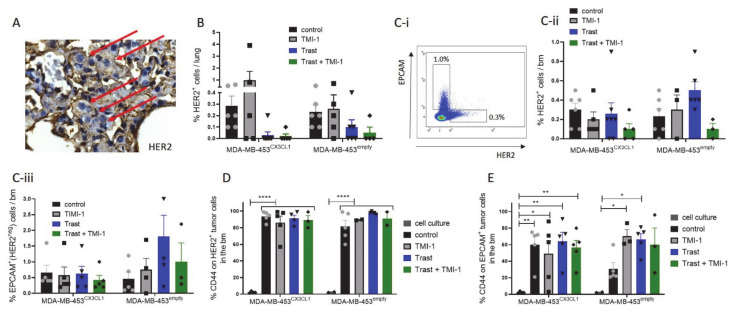
Metastases formation in MDA-MB-453-transplanted humanized tumor mice (HTM). **(A)** Immunohistochemical HER2 staining of a lung tissue exemplarily shown in a TMI-1-treated MDA-MB-453^CX3CL1^-transplanted HTM. Red arrows indicate tumor cells in the lung tissue. Metastases formation in the lung of HTM were analyzed by flow cytometry using an anti-HER2 antibody (**B**). (**Ci–iii**) Analysis of the bone marrow (bm) samples from the femurs revealed two different tumor cell populations (EPCAM^+^HER2^-^ and EPCAM^-^HER2^+^). CD44 expression on EPCAM^-^HER2^+^ (**D**) and EPCAM^+^HER2^-^(**E**) were analyzed using a multicolor panel. Data are shown as mean ± SEM, and significances were analyzed using Tukey’s multiple comparisons (* *p* < 0.05; ** *p* < 0.01; **** *p* < 0.0001).

All MDA-MB-453 transplanted control mice developed lung metastases (Figure 4A; Table 1), which could only be significantly reduced in trastuzumab-treated MDA-MB-453^CX3CL1^ (*p* = 0.0047) but not in MDA-MB-453^empty^-transplanted mice (Table 1). However, the combined trastuzumab/TMI-1 treatment significantly reduced lung metastases in both MDA-MB-453^CX3CL1^- and MDA-MB-453^empty^-transplanted mice (Table 1; *B *p* = 0.0152; *C *p* = 0.0333). Nevertheless, there was an individual and wide variation of tumor cell density in the lung of the analyzed HTM (Figure 4B).

Nearly all HTM showed HER2^+^ tumor cells in the bone marrow (bm), but neither trastuzumab nor TMI-1 treatment was able to completely eliminate these HER2^+^ disseminated tumor cells (DTCs) (Table 2; Figure 4C). Interestingly, an additional tumor cell population developed in the bm niche (which was not detectable in the lung) expressed EPCAM but lost HER2 on the cell surface (Figure 4C-i). This EPCAM^+^HER2^-^ DTC population represented the majority of DTC in all HTM (~1% of bm cells (Figure 4C-iii) compared to ~0.5% HER2^+^ DTCs (Figure 4C-ii)). HER2^+^ DTCs displayed similar to the analyzed primary tumor an increased CD44 expression compared to cell culture MDA-MB-453 (Figure 4D). This finding was independent of the applied treatments, whereas the increased CD44 expression in EPCAM^+^HER2^-^ DTCs exhibited higher variability in increased CD44 expression between treatments (Figure 4E).

### 3.5. MDA-MB-453^CX3CL1^-Transplanted HTM Developed Enlarged Spleens but Showed Similar High Human Immune Cell Reconstitution as MDA-MB-453^empty^-Transplanted Mice

**Figure 5:**
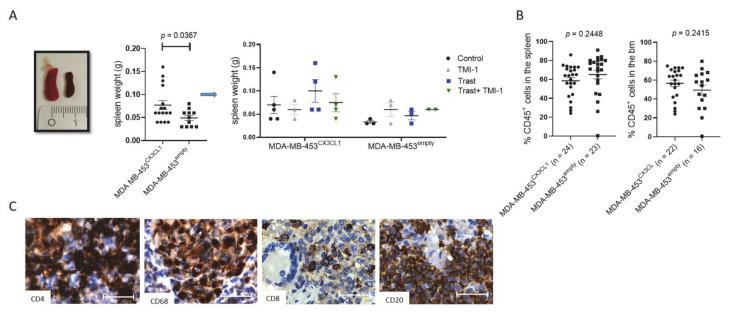
Human immune cell distribution in the spleen and bone marrow of MDA-MB-453^CX3CL1^ and MDA-MB-453^empty^ HTM. **(A)** Spleen weight and representative spleen image of MDA-MB-453^CX3CL1^ and MDA-MB-453^empty^ transplanted HTM is displayed and significance was calculated using Student’s *t*-test (*p* = 0.0367). (**B**) The percentages of human CD45^+^ immune cells in the spleen and bone marrow were analyzed using flow cytometry and differences were calculated using Student’s *t*-test. (**C**) Immune cell subsets in the spleen were stained with antibodies against CD4 (T helper cell), CD68 (macrophages), CD8 (cytotoxic T cell) and CD20 (B cell) antibody. Scale bar represents 50 μm.

Spleens were significantly enlarged in MDA-MB-453^CX3CL1^-transplanted mice (0.077 g ± 0.009 SEM; *n* = 16) compared to the spleen inspected in MDA-MB-453^empty^ HTM (0.049 g ± 0.005 SEM; *n* = 11¸ Figure 5A). Especially, four MDA-MB-453^CX3CL1^ HTM generated with four different cord blood donors showed an increased weight of this organ (one control, two trastuzumab and one trastuzumab + TMI-1 treated HTM; Figure 5A). At the age of eight weeks post HSC transplantation (before the animals were assigned to the experimental groups), mice were bled and the human reconstitution (% human CD45/live cells) was assessed. According to these results, humanized mice from the different donors were equally assigned to the respective groups (treatment and MDA-MB-453^empty^/^CX3CL1^). 

At the end of the experiments, spleen and bm cells were isolated and analyzed for human immune cell populations (gating strategy is shown in Supplementary Figure S5A,B). Both MDA-MB-453^CX3CL1^- and MDA-MB-453^empty^-transplanted mice showed high human reconstitution levels in the spleen (MDA-MB-453^CX3CL1^: 58.5 ± 3.4 SEM, *n* = 24; MDA-MB-453^empty^: 65.2 ±4.6 SEM; *n* = 23) and the bm (MDA-MB-453^CX3CL1^: 56.7 ± 3.4 SEM, *n* = 22; MDA-MB-453^empty^: 49.4 ± 5.3 SEM; *n* = 16) (Figure 5B). Immunohistological staining revealed a B cell (CD20) cluster formation in the spleen surrounded by CD68^+^ macrophages, which was accompanied by high numbers of CD4^+^ and lower number of CD8^+^ T cells (Figure 5C). There was a significant correlation between human immune cells in the blood (at the age of 8 weeks) and at the end of the experiment in the spleen (Pearson’s *r* = 0.5681; *p* < 0.0001; Supplementary Figure S5C). Therefore, the assignment of the humanized mice into the different groups (MDA-MB-453^empty^, MDA-MB-453^CX3CL1^ and treatment groups) based on the blood reconstitution at 8 weeks is feasible because it reflects the human immune cell reconstitution in the spleen, and therefore, the overall human immune cell repopulation in HTM. Notably, there were no differences in the overall human reconstitution or immune cell distribution between MDA-MB-453^CX3CL1^ and MDA-MB-453^empty^ in the spleen or BM (Supplementary Figure S5D,E). NK cells were analyzed in more detail (CD16, CD56 and CD27 expression), but no change in the overall percentage of NK cells (~1.3% of human cells) was found. In addition, no differentiation into the NK cell subpopulation (CD56/CD16) was measurable (the majority of NK cells showed CD56 expression (~65% in all groups), but a lower percentage showed CD16 expression (~36% in all groups). 

### 3.6. Rejection of Tumor Was Associated with Increased CD4^+^ T Cell Fraction and Maturation in HTM

A pretesting of cell engraftment in HTM using only 50,000 MDA-MB-453 tumor cells for s.c. transplantation yielded tumor outgrowth, though not in all transplanted mice. The immune cell analyses revealed a significant higher CD4 proportion in those animals without detectable tumor growth (Supplementary Figure S6B). Furthermore, CD4^+^ (Supplementary Figure S6C) as well as CD8^+^ (Supplementary Figure S6D) T cells showed an increased maturation stage (memory phenotype; gating strategy, Supplementary Figure S6A) in tumor-free HTM, suggesting an effective T cell defense against low tumor cell count transplantation.

**Figure 6:**
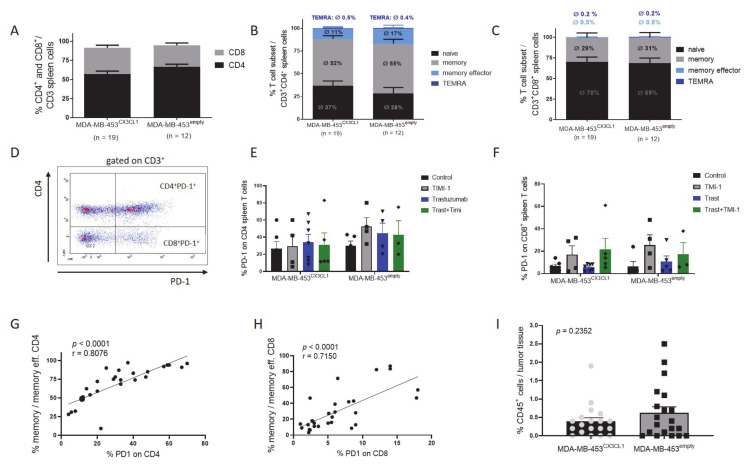
Human T cell subpopulation in the spleen upon transplantation with 1 × 10^6^ MDA-MB-453^CX3CL1^ and MDA-MB-453^empty^ cells into humanized mice. Humanized NSG mice were transplanted with 1 × 10^6^ MDA-MB-453^CX3CL1^ and MDA-MB-453^empty^ and spleen cells were analyzed by flow cytometry. The proportion of CD4^+^ and CD8^+^ T cells (**A**) and the different CD4^+^ (**B**) and CD8^+^ (**C**) subsets (naïve, memory (mem), memory effector, and terminally differentiated effector memory (TEMRA)) are displayed (mean of each subset is shown as average %). (**D**) PD-1 expression on CD4^+^ and CD8^+^ (CD4^−^) were analyzed. Differences in dependency of MDA-MB-453^CX3CL1^/MDA-MB-453^empty^ or treatment are displayed for CD4 (**E**) and CD8 (**F**) T cells. The correlation between PD-1 expression and a memory phenotype of CD4^+^ (**G**) and CD8^+^ (**H**) T cells was calculated (Pearson’s correlation coefficient *r* = 0.8076 and *r* = 0.7150). (**I**) The percentage of human immune cells (CD45^+^) infiltrating into the tumor tissue was analyzed (Student’s *t*-test *p* = 0.2352).

However, using 1 × 10^6^ tumor cells resulted in tumor engraftment in all transplanted mice. There were no significant differences in CD4/CD8% on T cells (Figure 6A and S6B) or maturation of CD4^+^ (Figure 6B and S6C) and CD8^+^ (Figure 6C and S6D) cells between MDA-MB-453^CX3CL1^ or MDA-MB-453^empty^. As previously found in HTM [24,26], CD4^+^ T cells were most often higher in number and showed advanced maturation compared to CD8^+^ T cells. Accordingly, PD-1 expression (Figure 6D gating strategy), as a marker for immune cell activation, was also higher in CD4 T cells (mean 35.3% CD4 ± 3.7 SEM; *n* = 38) compared to CD8 T cells (mean 12.67% CD8 ± 2.2 SEM; *n* = 39).

However, the CD4/CD8 proportion, the phenotypes and the PD-1 expression levels were independent of CX3CL1 overexpression and the applied treatments (Figure 6E,F and S6). Notably, there is a strong correlation between the maturation stage of T cells and PD-1 expression in CD4^+^ (Pearson’s *r* = 0.81; *p* < 0.0001; Figure 6G) and CD8^+^ (Pearson’s *r* = 0.72; *p* < 0.0001; Figure 6H) T cells, which strongly indicates that PD-1 expression is associated with an advanced maturation level in HTM. 

Analyses of the tumor tissue revealed an overall low immune cell infiltration (range: 0–2.5%; Figure 6I) in MDA-MB-453 transplanted HTM with a majority of T cells (MDA-MB-453^CX3CL1^ HTM: 66.4 ± 7.6 SEM; MDA-MB-453^empty^ HTM: 64.2 ± 7.2; data not shown) followed by CD33^+^ myeloid cells (MDA-MB-453^CX3CL1^ HTM: 18.1 ± 4.7 SEM; MDA-MB-453^empty^ HTM: 20.4 ± 5.8 SEM) and B cells (MDA-MB-453^CX3CL1^ HTM: 7.9 ± 4.7 SEM; MDA-MB-453^empty^ HTM: 8.5 ± 3.2, *n* = 15 for all groups). Again, these finding were independent of CX3CL1 overexpression and treatment modalities.

### 3.7. CX3CR1 Expression on Tumor Cells Was Significantly Increased In Vivo

**Figure 7:**
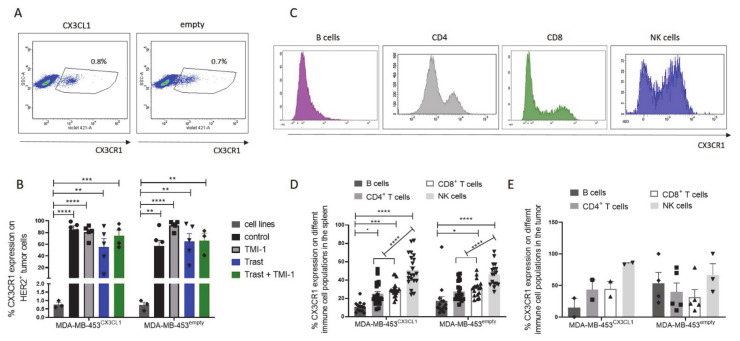
CX3CR1 expression on tumor and immune cells in vitro and in vivo. (**A**) Density dot plots of MDA-MB-453 (^CX3CL1^ or ^empty^ vector transfected) from cell culture stained with anti-CX3CR1 antibody. (**B**) CX3CR1 expression on MDA-MB-453^CX3CL1^ and MDA-MB-453^empty^ tumor cells isolated from the primary tumor of HTM compared with the same cells under cell culture conditions. (**C**) Histograms of CX3CR1 expression on B cells, CD4, CD8 and NK cells. Percentage of CX3CR1 expression on the different immune cells isolated from the spleen (**D**) and tumor (**E**) of MDA-MB-453-transplanted mice. Data are shown as mean ± SEM and Tukey’s multiple comparisons test was applied and significances are indicated (*p < 0.05; ** *p* < 0.01; *** *p* < 0.001; **** *p* < 0.0001).

CX3CR1 expression on tumor cells in cell culture were low, with an average of 0.8% CX3CR1^+^ tumor cells (±0.1 SEM; *n* = 6), which was independent of CX3CL1 expression (Figure 7A). However, in vivo CX3CR1 expression was significantly increased in all HTM but slightly affected by the different treatments (Figure 7B).

CX3CR1 expression was also detectable on different human immune cell populations in the HTM (exemplarily shown in Figure 7C) but independent of treatment or CX3CL1 overexpression (Figure 7D). The highest proportion of CX3CR1^+^ spleen cells were found on NK cells followed by CD4 and CD8 T cells (Figure 7D). B cells isolated from the spleen showed the lowest CX3CR1 content. Similar CX3CR1 profiles on the different immune cells were found on the tumor infiltrating immune cells (Figure 7E). 

## 4. Discussion

The importance of CX3CL1 in cancer biology is still being discussed and remains controversial. This might in part be due to the existence of two isoforms, i.e., a “soluble” and “membrane-bound” variant, and their different activities.

On the one hand, CX3CL1 seems to be associated with an enhanced immune cell infiltration into the tumor tissue, which contributes to a better outcome of, e.g., colorectal [32], gastric [33] and BC disease [22]. Moreover, membrane-bound CX3CL1 has been described to activate NK cell cytotoxicity [34], possibly triggered by an enhanced binding to the target cells [19]. However, in HTM, we did not detect CX3CL1-induced chemotactic function, which would be reflected by an increased immune cell tumor infiltration in CX3CL1 overexpressing MDA-MB-453 tumors, but we observed enlarged spleens in these mice. The low amount of immune cell infiltration observed in MDA-MB-453-based HTM seems to be due to the low immunogenicity of MDA-MB-453. Unlike MDA-MB-231-based HTM, HTM in general show a much higher degree of immune cell infiltration (unpublished data), and therefore, CX3CL1 expression might be more effective in these mice. However, as MDA-MB-231 cells do not express HER2, they were not suitable to study CX3CL1/trastuzumab synergies.

On the other hand, not only an anti-tumorigenic, but also a tumorigenic activity has been attributed to CX3CL1. For example, an enhanced formation of metastases in BC, especially to the bones, as a function of CX3CL1 has been reported [13,14,15]. However, this cannot be confirmed by the HTM experiments as performed in this study. There was no significantly enhanced formation of lung metastases or DTC accumulation in the bone marrow in MDA-MB-453^CX3CL1^ mice, even though the CX3CL1 receptor CX3CR1 was strongly upregulated in MDA-MB-453 tumor cells. Due to the limitation in single cell numbers that could be isolated from the lung and the bone marrow, CX3CR1 expression on lung and bone marrow tumor cells was not analyzed. However, we assume that the CXCR1 expression in vivo is generally increased, and therefore, appears to be expressed at the same density as seen in primary tumor cells.

In the solid tumor, we observed slightly enhanced tumor growth in CX3CL1 overexpressing tumors. An increased tumor growth under the influence of CX3CL1 has also been described for MDA-MB-453 and HT 29 transplanted SCID mice during the first period of engraftment (publication under review). The tumorigenic effect of CX3CL1 might be explained by the transactivation of EGFR downstream pathways that results in a stimulation of cell proliferation [17]. Notably, MDA-MB-453 tumor cells in vitro do not come with an EGFR gene amplification and do not express EGFR (analyzed by FISH and western blotting; unpublished data). Nevertheless, in MDA-MB-453-based HTM tumors, we found some degree of heterogeneity and EGFR positive tumor areas. Irrespective hereof, other authors described an enhanced tumor growth due to the stimulation of angiogenesis by CX3CL1 [35,36,37,38], which was not examined in HTM.

In this study, the predominant effect of CX3CL1 was the enhanced trastuzumab efficiency in MDA-MB-453^CX3CL1^-transplanted HTM. In vitro, trastuzumab treatment does not impair the viability of CX3CL1 overexpressing MDA-MB-453 or SK-BR-3 cells and does not significantly attenuate cell proliferation. In contrast, tumor growth of all MDA-MB-453-based HTM (MDA-MB-453^empty^ and MDA-MB-453^CX3CL1^) was significantly reduced by trastuzumab treatment. The therapeutic effect seems to be in part due to an immune cell activation that results in an immunological tumor defense, e.g., mediated by ADCC [7]. However, CX3CL1-expressing MDA-MB-453 HTM treated with trastuzumab showed a slightly enhanced inhibition of tumor growth indicated by an enhanced significance between control and trastuzumab treated HTM (MDA-MB-453^empty^ *p* = 0.0201versus MDA-MB-453^CX3CL1^ *p* > 0.0001) and a significantly reduced formation of lung metastases. This would confirm the assumption of CX3CL1 as a trigger for the activation of effector cells and promotor for stronger adhesion of leucocytes to CX3CR1-positive tumor cells as postulated before [19]. 

The CX3CR1 expression on different human immune cell subsets in HTM, predominantly on NK cells, supports this consideration. CX3CR1 expression was also found on activated cytotoxic T cells [39]. Correspondingly, we found in HTM human T cells with an upregulated PD-1 expression upon activation and maturation. These PD-1^+^/CD8^+^ infiltrating T cell have been characterized as tumor-specific and cytotoxic T cells in the clinical setting [40]. However, differences in T and NK cell maturation, or in PD-1 and CX3CR1 expression in spleen or tumor cells were not measurable between MDA-MB-453^empty^- and MDA-MB-453^CX3CL1^-based HTM or between the treatment groups. 

The application of the ADAM inhibitors TMI-1 as well as GI254023X blocked CX3CL1 shedding in vitro but without affecting the tumor cell viability or proliferation. TMI-1 mainly but not exclusively blocks ADAM17, a cell membrane located sheddase, which has more than 70 different substrates, amongst them CX3CL1, but also HER2, CD44, CD16, c-MET, MICA/B, EGFR, FASL and PD-L1 and growth factors such as TGF-α, amphiregulin, epiregulin and HB-EGF, which are released upon cleavage [41,42,43]. ADAM17 has been found overexpressed in various human cancers including BC [44] and has been associated with cancer progression, proliferation and migration [45], as well as with poor prognosis [46]. Therefore, the inhibition of ADAM17 might affect multiple tumor-relevant molecules and corresponding pathways. In the transgenic MMTV-ERBB2/neu mouse model, 30 days of TMI-1 treatment has been described to reduce tumor growth [47], but Tardáguila described a downregulation of CX3CL1 in the tumors of these mice compared to the healthy tissue. Moreover, the CX3CL1 transfection increased tumor formation in a dose-dependent manner [17]. Therefore, the role of TMI-1 in the context of CX3CL1 in this mouse model is difficult to determine. In MDA-MB-453^CX3CL1^–based HTM, TMI-1 treatment resulted in a slightly increased tumor growth in comparison to MDA-MB-453^empty^-based HTM, which suggests a pro-proliferative effect of membrane-bound CX3CL1 or the missing anti-proliferative effect of the soluble form. In summary, this study provides evidence for CX3CL1 contributing on the one hand to tumor growth and on the other hand to enhanced response to trastuzumab treatment in MDA-MB-453-transplanted HTM. However, an additional effect of ADAM inhibition could not be observed. In general, HTM are generated by using different cord blood donors in a combination of allogenic tumor cells, which might cause individual immune cell responses. Using different therapy modalities (e.g., long term TMI-1 substitution, increased concentration and others) might improve ADAM inhibitor efficacy but harbors the risks in term of side effects since ADAM enzymes are known to be expressed not only on target cells but on a variety of cells. GI254023X blocks mainly ADAM10 activity, which is responsible for the shedding of similar targets, including CX3CL1, EGFR, CD44 and HER2, as has also been described for ADAM17. Mullooly et al. described an impaired migration and invasion of MDA-MB-453 cells in vitro when exposed to GI254023X, but they also did not observe any effect on cell proliferation or survival [20]. In an in vivo orthotopic glioblastoma xenograft model, the application of GI254023X exhibited pronounced growth reduction [48]. However, the authors applied the inhibitor to block the cleavage and secretion of the synaptic adhesion molecule neuroligin-3 (NLGN3), and thereby, reduced its growth promoting function rather specific for glioma cells. Therefore, ADAM inhibition might act differently on individual tumor types depending on the molecules involved in proliferation or other tumor-specific pathways regulated by shedding.

A possible read-out parameter for treatment effectiveness in HER2^+^ BC in future HTM studies could be the degree of soluble HER2 ECD in the serum, which was reduced in trastuzumab-treated HTM and which correlated with a reduced tumor volume. The soluble ECD in the serum of patients has been also proposed to represent a marker for therapy response in humans [49]. The reduced serum ECD has also been described as a response marker to another ADAM inhibitor (i.e., INCB7839) tested in a single arm open label trial in combination with trastuzumab. Upon trastuzumab/INCB7839 administration, patients with HER2^+^ BCs showed a reduced concentration of ECD in the blood serum, and consequently, reduced constitutively active kinase p95-HER2, which was associated with an increased clinical response rate [50]. However, INCB7839 single-treated patients (without trastuzumab) were not included in this trial. The HTM experiments performed in this study revealed a reduced ECD concentration in the serum associated with reduced tumor size caused by trastuzumab treatment (with or without the application of TMI-1) but not in mice treated exclusively with TMI-1.

## 5. Conclusions

Overall, this study provides evidence for CX3CL1 contributing on the one hand to tumor growth and on the other hand to enhanced therapy response to trastuzumab treatment in MDA-MB-453-transplanted HTM. However, the inhibition of ADAMs by TMI-1 did not enhance the trastuzumab treatment success, neither in vitro nor in vivo, possibly hampered by the multifaceted effects of ADAM inhibitors. Nevertheless, the prognostic value of CX3CL1 expression in trastuzumab treated BC patients might be worth investigating. Once confirmed by the evaluation on human tumor samples, new approaches to enhance local CX3CL1 expression in combination with trastuzumab therapy should be tested in relevant pre-clinical models.

## Figures and Tables

**Table 1:** Detection of tumor cells in the lung of control versus treated HTM.

HER2^+^ Lung Metastases	MDA-MB-453^CX3CL1^ (*n*/*n*)	MDA-MB-453^empty^ (*n*/*n*)
Control	6/6	6/6
TMI-1	3/5	4/5
Trast	1/7 *^A^	3/6
Trast and TMI-1	1/5 *^B^	1/4 *^C^

Single cells from the lung were analyzed by flow cytometry. The number of animals with detectable HER2^+^ tumor cells of the total number of animals (*n*/*n*) is given. Statistical differences were calculated using the two-sided Fisher’s exact test and significant differences between control and trastuzumab (Trast; *A *p* = 0.0047) or control versus trastuzumab + TMI-1 (*B *p* = 0.0152; *C *p* = 0.0333) are indicated in the table.

**Table 2:** Detection of tumor cells in the bm of control versus treated HTM.

HER2^+^ bm Metastases	MDA-MB-453^CX3CL1^ (*n*/*n*)	MDA-MB-453^empty^ (*n*/*n*)
Control	6/6	5/6
TMI-1	5/5	2/3
Trast	5/7	6/6
Trast and TMI-1	3/5	2/3

Single cells from the femur were analyzed by flow cytometry and HTM with detectable HER2^+^ tumor cells were counted. The number of animals with detectable HER2^+^ tumor cells of the total number of animals (*n*/*n*) is given. Statistical differences were calculated using the two-sided Fisher’s exact test and revealed no significant differences.

## Supplementary Materials

The following are available online at https://www.mdpi.com/article/10.3390/cancers13102459/s1, Figure S1: Timeline for mouse transplantation and treatments, Figure S2: Treatment efficiency of trastuzumab and TMI-1 on SK-BR-3 tumor cells, Figure S3: Efficiency of trastuzumab and TMI-1 treatment on MDA-MB-453 cells, Figure S4: Treatment efficiency of trastuzumab and GI254023X treatment on MDA-MB-453 cells in vitro, Figure S5: Human immune cell reconstitution in MDA-MB-453^CX3CL1^- and MDA-MB-453^empty^-transplanted HTM, Figure S6: CD4^+^ and CD8^+^ T cell maturation in HTM transplanted with a reduced number of MDA-MB-453^CX3CL1^- and MDA-MB-453^empty^ cells, Table S1: Overview of tumor load and metastasized tumor cells in the lung and bone marrow of individual MDA-MB-453^CX3CL1^- and MDA-MB-453^empty^-transplanted HTM.
