# Supplementary material for: CX3CL1 Overexpression Prevents the Formation of Lung Metastases in Trastuzumab-Treated MDA-MB-453-Based Humanized Tumor Mice (HTM)
Source: Cancers (Basel). 2021 May 18;13(10):2459. doi: 10.3390/cancers13102459 (PMC8158361; doi:10.3390/cancers13102459)
Supplement: Supplementary file 1 [file cancers-13-02459-s001.zip › cancers-1161296-supplementary.pdf]

cancers

MDPI

Supplementary Materials

CX3CL1 Overexpression Prevents the Formation of Lung Metastases in Trastuzumab-Treated MDA-MB-453-Based Humanized Tumor Mice (HTM)

Anja K. Wege, Tobias F. Dreyer, Attila Teoman, Olaf Ortmann, Gero Brockhoff and Holger Bronger

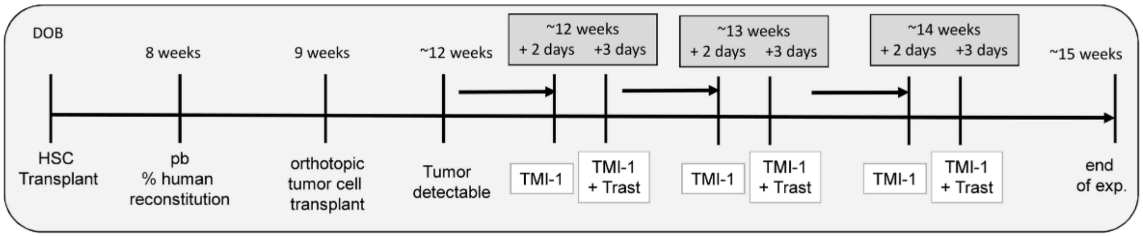

Figure S1. Timeline for mouse transplantation and treatments. Neonatal NSG mice were transplanted with $CD34^+$ hematopoietic stem cells. Eight weeks later humanized mice have been bled and analyzed for the presence of human immune cells (immune cell reconstitution). Mice that show more than 20% $hCD45^+$ cells in the peripheral blood were transplanted with MDA-MB-453 BC cells in the mammary fad pad. Two and three days after tumor detection (~12 weeks of age) TMI-1 treatment (100 mg/kg) started and were combined with trastuzumab (5 mg/kg) on day 3 post tumor detection. This strategy was repeated for 3 weeks and 1 week after the last treatment HTM were analyzed (~15 weeks of age). (DOB = day of birth; HSC = hematopoietic stem cells).

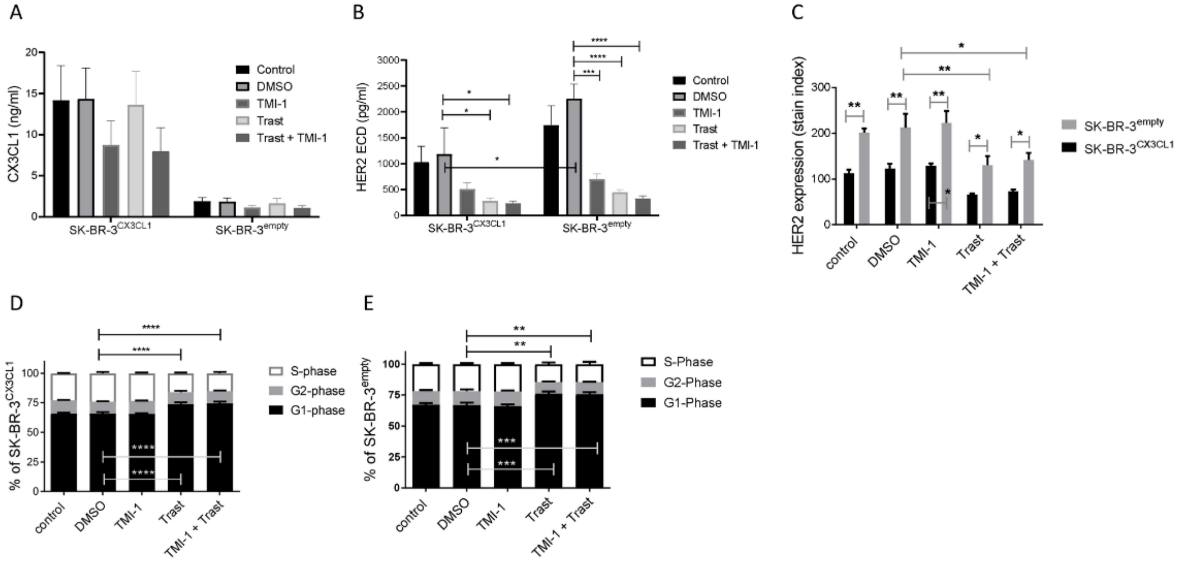

Figure S2. Treatment efficiency of trastuzumab and TMI-1 on SK-BR-3 tumor cells. SK-BR-3^{CX3CL1} and SK-BR-3^{empty} tumor cells were treated with DMSO, TMI-1, trastuzumab (Trast) or trastuzumab + TMI-1 for 48 h. Supernatants of the treated cell cultures were analyzed for CX3CL1 (**A**) or the extracellular domain of HER2 (HER2 ECD; **B**) in comparison to DMSO treated control cells. (**C**) HER2 expression intensity of the treated and untreated SK-BR-3 cells was analyzed by flow cytometry and stain index [MFI = mean fluorescence intensity; stain index = (MFI Her2–MFI Isotype)/(2 (x) SD Isotype)] was calculated. (**D** & **E**) Apoptosis induction was measured by flow cytometry using annexin and DAPI to allow the differentiation of live ($Annexin^- DAPI^-$) early apoptotic ($Annexin^+ DAPI^-$), late apoptotic ($Annexin^+ DAPI^+$), or very late apoptotic ($Annexin^- DAPI^+$) cells. All experiments were performed three times. Data are shown as mean ± SEM and Dunnett's multiple comparisons test (A, B, D, W) or Sidak's multiple comparisons test (**C**) was applied and significances indicated (* $p < 0.05$; ** $p < 0.01$; *** $p < 0.001$; **** $p < 0.0001$).

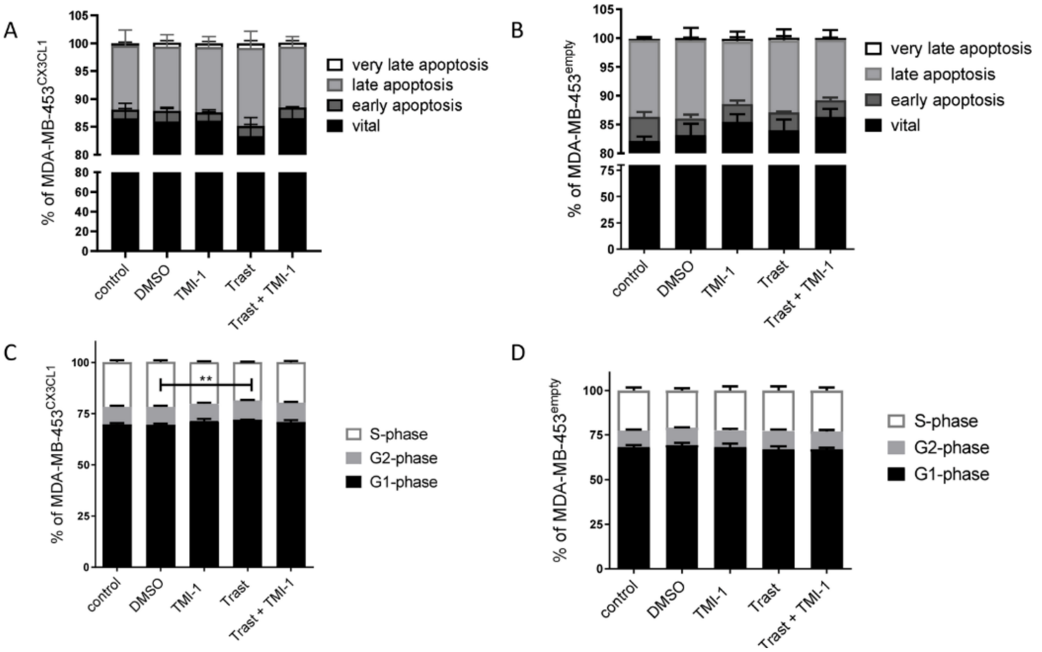

Figure S3. Efficiency of trastuzumab and TMI-1 treatment on MDA-MB-453 cells. MB453^{CX3CL1} (**A**, **C**) and MDA-MB-453^{empty} (**B**, **D**) tumor cells were treated with DMSO, TMI-1, trastuzumab (Trast) or trastuzumab + TMI-1 for 48 h. (**A&B**) Apoptosis induction was measured by flow cytometry using annexin and DAPI to allow the differentiation of live (Annexin$^-$ DAPI$^-$), early apoptotic (Annexin$^+$ DAPI$^-$), late apoptotic (Annexin$^+$ DAPI$^+$), or very late apoptotic (Annexin$^-$ DAPI$^+$) cells. (**C&D**) S-phase, G1, and G2 fraction were analyzed by flow cytometry. All experiments were performed three times. Data are shown as mean ± SEM and Tukey's multiple comparisons test was applied and significances indicated (* $p < 0.05$; ** $p < 0.01$).

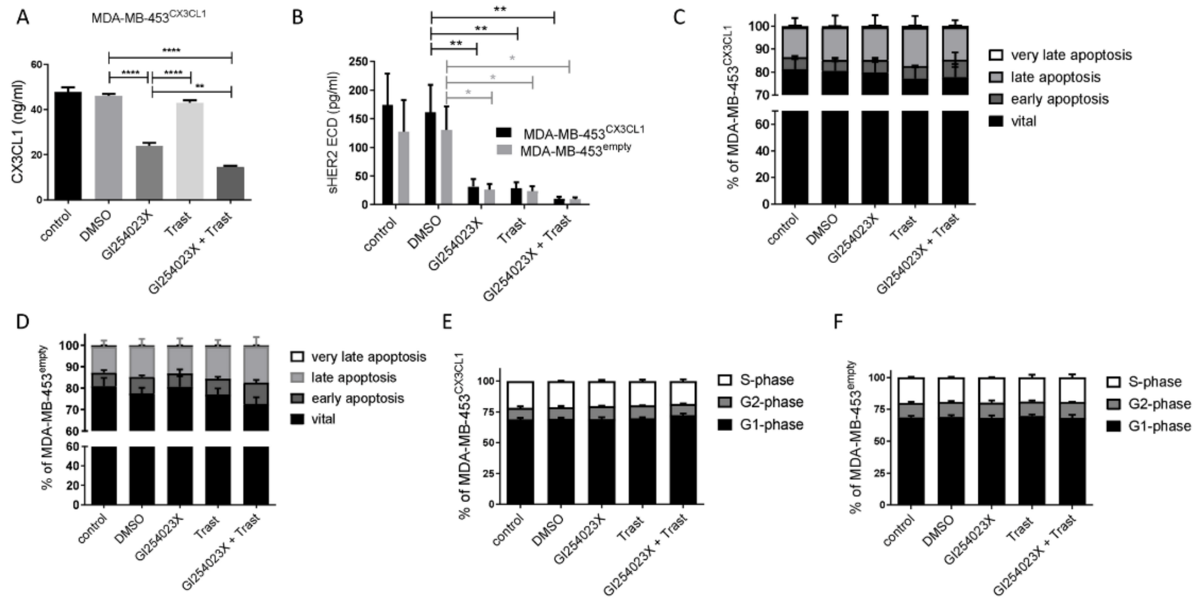

Figure S4. Treatment efficiency of trastuzumab and GI254023X in-vitro. MDA-MB-453^{CX3CL1} and MDA-MB-453^{empty} tumor cells were treated with DMSO, GI254023X, trastuzumab (Trast) or trastuzumab + GI254023X for 48 h. Supernatants of the treated cell cultures were analyzed for CX3CL1 (**A**) or the extracellular domain of HER2 (HER2 ECD) in comparison to DMSO treated control cells. (**C&D**) Apoptosis induction was measured by flow cytometry using annexin and DAPI to allow the differentiation of live (Annexin$^-$ DAPI$^-$) early apoptotic (Annexin$^+$ DAPI$^-$), late apoptotic (Annexin$^+$ DAPI$^+$), or

very late apoptotic (Annexin$^-$ DAPI$^+$) cells. (**E&F**) S-phase, G1, and G2 fraction were analyzed by flow cytometry. All experiments were performed three times. Data are shown as mean ± SEM and Tukey's multiple comparisons test was applied and significances indicated (* $p < 0.05$; ** $p < 0.01$; **** $p < 0.0001$).

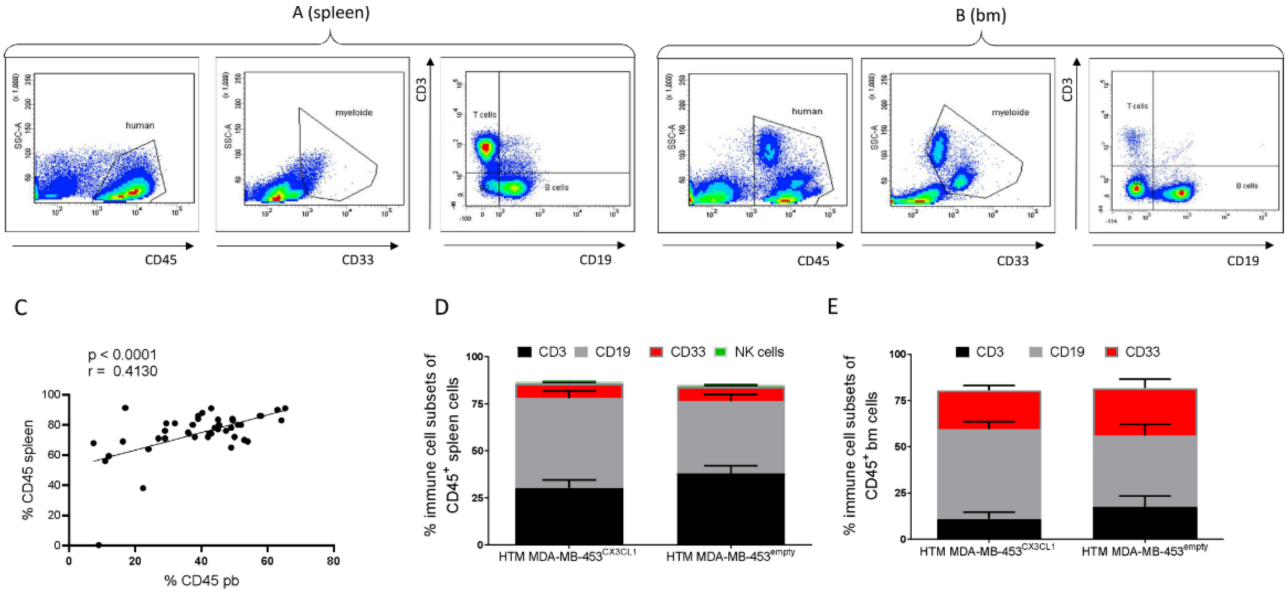

Figure S5. Human immune cell reconstitution in MDA-MB-453 and MDA-MB-453 transplanted HTM. Immune cell gating strategies for the spleen (**A**) and the bone marrow (BM; **B**) are displayed. (**C**) The correlation of the reconstitution levels (% CD45 cells)taken at the age of 8 weeks from the peripheral blood (pb) and from the spleen at the end of the experiments were calculated (Pearson's correlation coefficient r = 0.4130). The overall human reconstitution (% human immune cells; CD45) in all analyzed MDA-MB-453 and MDA-MB-453 transplanted HTM in the spleen (**D**) and bone marrow (bm; **E**) were analyzed by flow cytometry.

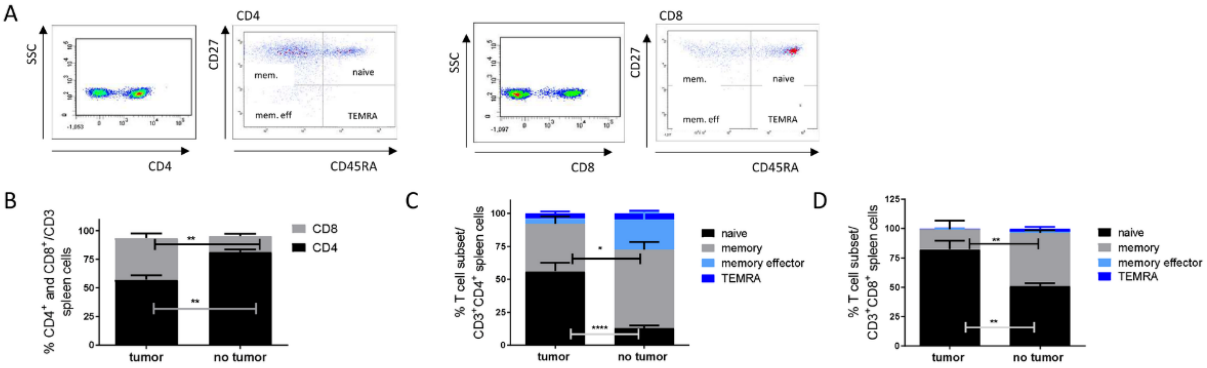

Figure S6. CD4$^+$ and CD8$^+$ T cell maturation in HTM transplanted with a reduced number of MDA-MB-453^{CX3CL1} and MDA-MB-453^{empty} cells. Humanized NSG mice were transplanted with only 50.000 MDA-MB-453CX3CL1 and MDA-MB-453empty cells, and HTM with tumors were compared to HTM without tumor development. (**A**) Gating strategy for the detection of CD4$^+$ and CD8$^+$ subsets (naïve, memory (mem), memory effector (mem. Eff.), and terminally differentiated effector memory (TEMRA). HTM with tumors were compared to HTM without tumor development showing differences in the CD4$^+$/CD8$^+$ proportion (**B**) and the T cell sub populations of CD4$^+$ (**C**) and CD8$^+$ (**D**) T cells isolated from the spleen. Significances were calculated using Sidak's multiple comparisons test (* $p < 0.05$; ** $p < 0.01$; **** $p < 0.0001$).

Table S1. Overview of tumor load and metastasized tumor cells in the lung and bone marrow of individual MDA-MB-453^{CX3CL1} and MDA-MB-453^{empty} transplanted HTM. Tumor load of each MB453^{CX3CL1} and MDA-MB-453^{empty} transplantated HTM was calculated (Volume (mm3) = (l × w^2)/2) at the end of the experiment and single cells isolated from lung and bone marrow were analzed by flow cytometry to define % of HER2+ tumor cells. DTC = disseminated tumor cells in the bone marrow; n.d.= no data¸*early termination of the experiment due to animal welfare reasons. ** mouse died.

MDA-MB-453^{CX3CL1}				
**Mouse #**	**Treatment**	**Tumor Volume (mm^3)**	**Lung Metastases (% HER2$^+$ Tumor Cells)**	**DTC (% HER2$^+$ Tumor Cells)**
HUM 7	control	269,5	0.1	0.1
HUM 22	control	567	0.2	0.4
HUM 32	control	162*	0.1	0.4
HUM 53	control	320	0.6	0.3
HUM 36	control	245	0.5	0.1
HUM 48	control	180	0.2	0.5
HUM 15	TMI-1	137.5	0	0.2
HUM 17	TMI-1	198	3.9	0.1
HUM 35	TMI-1	416	0.8	0.1
HUM 38	TMI-1	1224	0.2	0.5
HUM 25	TMI-1	61	0	0.1
HUM 9	Trast	18	0	0
HUM 16	Trast	0	0	0
HUM 23	Trast	8	0.2	0.2
HUM 31	Trast	112.5*	0	0.9
HUM 37	Trast	1	0	0.3
HUM 44	Trast	40	0	0.2
HUM 52	Trast	0	0	0.2
HUM 14	TMI-1 +Trast	27	0.1	0
HUM 18	TMI-1 +Trast	16	0	0
HUM 20	TMI-1 +Trast	0	0	0.1
HUM 33	TMI-1 +Trast	0	0	0.1
HUM 39	TMI-1 +Trast	1	0	0.3
MDA-MB-453^{empty}				
**Mouse #**	**Treatment**	**Tumor Volume (mm^3)**	**Lung Metastases (% HER2$^+$ Tumor Cells)**	**DTC (% HER2$^+$ Tumor Cells)**
HUM 10	control	144	0.1	0.4
HUM 19*	control	112.5	0.2	0
HUM 30	control	270	0.3	0.5
HUM 45	control	196	0.5	0.1
HUM 47	control	320	0.2	0.1
HUM 51	control	608	0.1	0.3
HUM 13	TMI-1	623	0.1	0.5
HUM 27	TMI-1	48	0.7	0
HUM 28	TMI-1	269.5	0.3	n.d.
HUM 41	TMI-1	180	0.2	0.4
HUM 20-27	TMI-1	56	0	
HUM 25	Trast	0	0.1	n.d.
HUM 34	Trast	0	0	0.4
HUM 29	Trast	0,5	0	0.9
HUM 43	Trast	18	0	0.4
HUM 40	Trast	40	0.1	0.4
HUM 8	Trast	62.5	n.d.	0.6
HUM 49	Trast	0	0.4	0.3
HUM 24	TMI-1 +Trast	6	0.2	n.d.
HUM 26	TMI-1 +Trast	13.5	0	0
HUM 42	TMI-1 +Trast	13.5	0	0.2
HUM 11	TMI-1 +Trast	18	**	**
HUM 31	TMI-1 +Trast	0	0	0

DTC = disseminated tumor cells in the bone marrow¸*early termination of the experiment due to animal welfare reasons. DTC = disseminated tumor cells in the bone marrow; n.d.= no data¸*early termination of the experiment due to animal welfare reasons. **mouse died.
